# Fluorescence ImmunoPrecipitation (FLIP): a Novel Assay for High-Throughput IP

**DOI:** 10.1186/s12575-016-0046-x

**Published:** 2016-08-15

**Authors:** Paolo Mita, Tenzin Lhakhang, Donghui Li, Daniel J. Eichinger, David Fenyo, Jef D. Boeke

**Affiliations:** 1 Institute of Systems Genetics (ISG), Department of Biochemistry and Molecular Pharmacology, NYU Langone Medical Center, ACLSW Room 560, 430 East 29th Street, New York, NY 10016 USA; 2 Center for Health Informatics and Bioinformatics, and Department of Biochemistry and Molecular Pharmacology, NYU Langone Medical Center, New York, NY USA; 3 McKusick-Nathans Institute of Genetic Medicine, Johns Hopkins University School of Medicine, Baltimore, MD 21205 USA; 4 High Throughput Biology Center, Johns Hopkins University School of Medicine, Baltimore, MD 21205 USA; 5 CDI Labs, Inc., Mayaguez, Puerto Rico

**Keywords:** Immunoprecipitation, Immunoblotting, High-throughput, HuEV-A vector, Antibodies, Screening

## Abstract

**Background:**

The immunoprecipitation (IP) assay is a valuable molecular biology tool applied across a breadth of fields. The standard assay couples IP to immunoblotting (IP/IB), a procedure severely limited as it is not easily scaled for high-throughput analysis.

**Results:**

Here we describe and characterize a new methodology for fast and reliable evaluation of an immunoprecipitation reaction. FLIP (FLuorescence IP) relies on the expression of the target protein as a chromophore-tagged protein and couples IP with the measurement of fluorescent signal coating agarose beads. We show here that FLIP displays similar sensitivity to the standard IP/IB procedure but is amenable to high-throughput analysis. We applied FLIP to the screening of mouse monoclonal antibodies of unknown behavior in IP procedures. The parallel analysis of the considered antibodies using FLIP and IP/western shows good correlation between the two procedures. We also show application of FLIP using unpurified antibodies (hybridoma supernatant) and we developed a publicly available tool for the easy analysis and quantification of FLIP signals.

**Conclusions:**

Altogether, our characterizations of this new methodology show that FLIP is an appealing and reliable tool for any application of high-throughput IP.

**Electronic supplementary material:**

The online version of this article (doi:10.1186/s12575-016-0046-x) contains supplementary material, which is available to authorized users.

## Background

In the post-Human Genome Project era, increasing and growing interest is devoted towards proteomic approaches aimed at gaining new insight into the complex ways cells interpret and regulate genomic information. Consortia such as the Encyclopedia of DNA Elements consortium and the Human Proteome Organization (HPP) aim to fill the gap between the genomic and the proteomic world [1, 2]. Mass spectrometry and antibody capture analyses are the basic experimental strategies used towards this goal. An essential tool for the study of the function and interaction of proteins is the immunopurification of the protein of interest with or without its interacting partners and molecules [3, 4].

The immunoprecipitation assay (IP) is an important tool used in academic and industrial research labs for applications such as targeted protein purification, protein concentration, analysis of protein-protein interactions, identification/analysis of protein complexes and analysis of protein/DNA interactions using chromatin IP (ChIP). IP and ChIP are both inexpensive and highly informative, relying on a specific antibody to selectively bind to the target epitope with high affinity [5]. By combining this binding reaction with a high molecular weight entity such as a bead, a bacterial cell or a meshwork of secondary antibodies, it is possible to isolate the protein of interest and its binding partner(s) from all the other cellular components in a microfuge tube [6]. However, not all antibodies are well-suited for IP. For instance, for some IP applications the antibody should recognize the target protein in its three-dimensional folded state, which requires that the epitope be exposed on an accessible surface of the target protein [7]. Therefore, there is a demand for high-throughput assays to screen antibodies capable of IP of target proteins [8, 9]. Moreover, because of the wide range of applications of the IP assay, quick and high-throughput ways to determine the success of an IP are necessary. Until now the standard procedure to validate antibodies for IP couples the IP assay to immunoblotting (IP/IB), a procedure that is challenging to scale to high-throughput analysis [10]. Novel approaches for antibody validation combine immunoprecipitation with mass spectrometry analysis (IP/MS) but, despite the quantitative, highly sensitive and informative nature of this approach, IP/MS is still a more expensive and labor- and instrument-intensive procedure compared to the more accessible IP/IB [6, 11]. Moreover, success of IP/MS is highly dependent on both the cell type and the abundance of the protein of interest.

To our knowledge the only high-throughput assay developed for IP screening is LUMIER (luminescence-based mammalian interactome mapping) system [12, 13]. LUMIER relies on the quantification of the luminescence signal produced by the immunoprecipitation or co-immunoprecipitation of an over-expressed target protein tagged with Renilla luciferase. LUMIER was successfully applied to a range of applications but, to our knowledge, is still the only high-throughput option to classical IP/WB.

We have developed a novel assay for immunoprecipitation, termed FLuorescence IP (FLIP) that is quick, reliable, easy to perform and scalable because it does not rely on immunoblotting analysis. Additionally, due to the small amount of material required, the FLIP assay can be included in a standard IP/IB procedure without affecting the output of the analysis. Because of these features, the FLIP assay is ideal for high-throughput screening to identify IP-grade antibodies. Here we describe the FLIP concept and apply it to the high-throughput identification of IP-proficient mouse monoclonal antibodies.

## Methods

### HuEV-A Cloning and Cre Recombination Reaction

The HuEV-A vector was constructed from a one-step ligation reaction with two inserts and a vector fragment. The backbone for the HuEV-A vector was the previously described pCEP4-Puro plasmid [14]. The first insert was a DNA fragment containing the chloramphenicol resistance gene, ccdB gene and att-R2 site, and was constructed by joining a *Not*I/*Kpn*I segment of the Gateway Reading frame A cloning vector (Life Technologies, Cat #11828-029) containing the chloramphenicol resistance gene and the att-R2 site to a second synthetic DNA fragment (UEV) containing the complementary att-R1 site along with a series of epitope tags, recombination sites and the Venus YFP sequence with *Bsi*WI (compatible with *Bsr*GI) and *Eag*I (compatible with *Not*I) termini. The pCEP Puro backbone vector was digested with *Kpn*I and *Bsr*GI and the inserts were ligated into the backbone using T4 DNA ligase in ligase buffer (Enzymatics, Beverly, MA; Prod. No L6030-HC). Vectors were transformed into *E. coli* and selected on ampicillin/chloramphenicol plates. The HuEV-A vector has been deposited at Addgene (plasmid number 68342).

Cre recombination reactions were performed to generate HuEV-A expression plasmids lacking YFP in the tag. One unit of Cre recombinase (New England Biolabs, M0298), 0.25 μg of HuEV-A plasmid with intact tag, was incubated in 50 μl of 1X Cre buffer (New England Biolabs, Beverley, MA) at 37 °C for 30 min. Cre recombinase was then inactivated for 10 min at 70 °C and the DNA purified on column (Qiagen, 28106). The eluted DNA was then cut with *PmlI* enzyme for 1 h at 37 °C in CutSmart buffer (New England Biolabs) to cut the not recombined plasmids. *PmlI* has a unique site in the sequence between the LoxP sites. DNA was again column purified, transformed in ccdB resistant competent cells (Invitrogen, Carlsbad, CA; Prod. No A10460) and plated on ampicillin selection plates. Colonies were screened by colony PCR and the chosen clones verified by sequencing.

### Cell Culture and Transient Transfection

HeLa-M2 cells were cultured in DMEM supplemented with 10 % FBS (Gemini, Prod. No 100–106) and 1 mM L-glutamine (ThermoFisher/Life Technologies, Prod. No 25030–081) [15]. Cells were routinely split in fresh medium and new plates upon reaching 80–90 % confluence. During routine culture of the cells the medium was changed every 2–3 days.

The day before transfection 0.3 × 10^6^ cells were plated in each well of a 6-well plate (Falcon). The day after plating, transfection was performed using Fugene-HD reagent according to the manufacturer’s instructions (Promega, Madison, WI; Prod. No E2311). For each well, 0.75 μg of DNA, 3 μl Fugene-HD and 50 μl of Opti-MEM (Life Technologies, Prod. No 31985–070) were incubated 15 min at room temperature. The transfection mixture was then dripped into each well containing 2 ml of complete media and the HeLa M2 cells plated the previous day. Transfection conditions (number of cells plated, DNA amount and Fugene-HD amount) used in Fig. 4 are reported in Additional file 1: Figure S4. We found that high transfection efficiency (>80 %) depended on (i) confluency of cells prior to plating (cell density >80 % confluence was detrimental); (ii) duration of incubation of plated cells prior to introduction of foreign DNA/Fugene (incubation after plating of >18 h was required); (iii) time after transfection before doxycycline treatments (doxycycline treatments immediately after transfection induced much lower expression compared to doxycycline treatments started 18–24 h after transfection); and purity/quality of DNA (all plasmids were prepared by Maxi or Midipreps [Invitrogen PureLink, plasmid filter kits, product number K210015]).

For the experiments presented here, doxycycline was added to the medium immediately after transfection at a final concentration of 1 μg/ml, which limited protein expression from the HuEV-A vector but shortened the timeline to cell harvest by a day. The expression of the desired protein under the indicated conditions was sufficient to guarantee a reliable FLIP and/or an IP/IB analysis in 96 % of the cases. Sporadic low expression was attributed to inaccurate cloning of the target protein into the expression vector.

### Cell Lysis and Conventional Immunoprecipitation

For the parallel analysis of mouse antibodies (CDI Laboratories) using FLIP and IP/IB assays in a 96 well IP format (Fig. 5), an entire 6-well plate was used for each target protein. Cells were treated with 0 (one well), 50 ng/ml (2 wells) or 1 μg/ml (3 wells) doxycycline and lysates obtained from one well were used for one IP. After 24 h of doxycycline treatment, cells were lysed in Triton lysis buffer (20 mM HEPES, 1 % Triton X-100, 500 mM NaCl) supplemented at the time of use with protease inhibitor tablets (Roche, Prod. No 11697498001). 150 μl of lysis buffer were used to lyse the cells cultured in one well, and 120 μl of clarified cell lysate were used for each IP/ FLIP. IPs were performed with a normal mouse IgG Abs (Sigma, Prod. No sc-2025) as a negative control (using lysates from cells treated with 1 μg/ml doxycycline); FLAG-M2 antibody (Sigma, Prod. No F1804) as positive control (using lysates from cells treated with 1 μg/ml doxycycline); or the test antibody (using lysates from cells treated with 50 ng/ml and 1 μg/ml doxycycline). After SDS-PAGE and transfer of the proteins onto a PVDF-F membrane, proteins were probed with a rabbit polyclonal antibody against FLAG tag (Cell Signaling #2368) mixed with the test mouse antibody. Secondary goat antibodies conjugated to IRDye680 (anti-rabbit) or IRDye800 (anti-mouse) dyes (LiCOR), were used for the detection of the recognized bands on an Odyssey CLx scanner (LiCOR). The efficiency with which the tested mouse Ab was able to immunoprecipitate the over-expressed target protein was calculated by quantifying the intensity of the band of the protein immunoprecipitated with the tested mAb over the intensity of the band of the protein from the total lysate lane (IP INPUT) (Additional file 1: Figure S5). Correcting for the different volumes/amount loaded on the gel, the % of target protein immunoprecipitated (% tot. lysate) was calculated. FLIP was performed on the beads from FLAG IP, IgG IP and tested mAb IP and using only cell lysates from cells treated with 1 μg/ml doxycycline.

For the experiments described in Fig. 3, HeLa cells were plated and transfected in multiple 3-cm wells as described above, and all wells were treated with 1 μg/ml doxycycline for 24 h starting from the day after transfection. Cells were lysed in Triton buffer supplemented with protease inhibitors, and FLIP was performed as described below in 1.5 ml tubes (low throughput FLIP).

For the IP performed with hybridoma supernatant (Fig. 7), lysates from cells expressing UPF1 protein from the HuEV-A vector were used. Lysates from about two 3-cm wells were used to IP the tagged UPF1 protein using 200 μl (≈1:3 dilution) of hybridoma supernatant, (of unknown antibody concentration), or control mouse IgG antibodies (5 μg). The IP was conducted in 0.7 ml of total volume in 1.5 ml tubes using protein A/G beads [16, 17].

A Mini-FLIP assay (described below) used to evaluate the same hybridoma supernatants was performed using 40 μl (1:3 dilution) of hybridoma supernatant in a total volume of 120 μl.

### FLIP

Before performing FLIP assays, the YFP content of lysates from cells expressing the YFP-tagged protein of interest was measured to determine the amount of YFP present in each μl of lysate. The fluorescence of 10 μl of lysate was measured in clear-bottom black 384-well plates and compared to a standard curve obtained measuring the fluorescence of known amounts of recombinant YFP (MBL International Corporation; Prod. No JM-4998-100) (a typical standard curve ranged from 0 to 500 ng of recombinant YFP). If an autogain was set for the fluorescence reading, the samples and the standard curve dilutions were always read on the same plate. In this study, an excitation wavelength of 475 nm, and emission wavelength of 527 nm, were used for YFP detection using a Synergy H1 reader (BioTek). After quantifying the amount of YFP, an estimate of FLIP efficiency was determined from the curve in Fig. 3a. Considering that the curve in Fig. 3a was obtained using a well-behaved FLAG antibody, 700 μl of lysate containing at least 0.5 ng/μl of YFP (at least 350 ng of total YFP per IP) should be used for each FLIP to ensure a reliable measurement and quantification of fluorescence from the beads using FLIP performed in 1.5 ml tubes (large volume FLIP). Our characterizations showed that the sensitivity of the FLIP increased with an increase of YFP-protein in the lysate used for the assay. To ensure reliable FLIP results, especially for antibodies with weak affinity, the use of more than 350 ng of YFP-protein in the initial lysate is recommended.

A much lower amount of YFP and lysate is needed for mini-FLIP assays, in which 100–120 μl of lysate containing at least 0.3 ng/μl of YFP-protein can be used.

We have observed the YFP fluorescence on the beads to be highly stable in our FLIP assays. YFP signal coating the agarose beads was still observed up to one week after IP, with a negligible decrease in FLIP sensitivity, when the plate containing the FLIP beads was stored in the dark at 4 °C.

### Large Volume FLIP

Lysates from cells expressing the YFP-protein of interest were aliquoted into 1.5 ml tubes (700 μl per tube). 5 μg of tested or control IgG antibodies was added directly into the lysates and incubated for 1 h at 4 °C on a nutating mixer (Fisher Scientific) to ensure continuous agitation of the sample. 50 μl of Protein A/G agarose beads (Santa Cruz, Prod. No sc-2003) slurry was then added to each tube and incubated for 30 min at 4 °C on a roller. Beads were washed 5 times in Triton buffer (800 μl of buffer per wash). During the last wash, 10 μl of beads/buffer solution were transferred into black 384 well plates for analysis with a microscope, while the remainder of the beads were collected and processed for immunoblotting analysis. For the latter analysis, the immune complexes were eluted from the beads by adding 1X NuPage LDS sample buffer (Life Technologies, Prod. No NP0007) supplemented with 350 mM β-mercaptoethanol. Samples were heated at 75 °C for 10 min and stored at −20 °C until immunoblotting analysis.

### High Throughput (HiT) FLIP

For the parallel analysis of mouse monoclonal antibodies using FLIP and IP/IB assays presented in Fig. 5, a 96-well format was used for IP/FLIP. 700 μl of lysates prepared as described above were aliquoted to each well of a 96-deep-well plate (Nunc). The 96-well format allowed for manipulation of the samples using multichannel pipettes. Each YFP-protein expressed in HeLa cells was treated with 0, 50 or 1000 ng/ml doxycycline as described above. 5 μg of antibodies was added to the appropriate wells containing the target YFP-protein of interest. Every antibody required 4 wells for analysis: mouse IgG control Ab was added to a well containing lysates of cells treated with 1000 ng/ml doxycycline; anti FLAG-M2 Ab was added to a well containing lysates of cells treated with 1000 ng/ml doxycycline; mAb to be tested was added to a well containing lysates of cells treated with 1000 ng/ml doxycycline and to a well containing lysates of cells treated with 50 ng/ml doxycycline. The plate was sealed with aluminum sealing tape and incubated 1 h at 4 °C on a nutator to ensure continuous agitation of the sample. 50 μl of Protein A/G agarose bead slurry were added to each well and incubated for 30 min at 4 °C on a nutator. Beads were then washed 5 times with Triton buffer without disturbing the bead pellet when removing the supernatant. Approximately 50–100 μl of Triton buffer was left in each well during washes, and long loading tips (Fisher) were used to discard the washes to minimize bead loss. Multichannel tip adaptors attached to a vacuum pump were used for quick handling of the samples during washes. After the last wash (with 800 μl), 10 μl of beads from the mouse IgG IP and from the IP with the tested Ab was collected from each well and transferred to a 384 well plate. These samples were then analyzed for FLIP while the rest of the beads were processed for immunoblotting analysis. The left-over beads were collected by centrifugation and the last wash was discarded using loading tips. The bead pellet can be quickly touched to get rid of most of the buffer before eluting the immuno-complexes into 1X NuPage LDS sample buffer supplemented with 350 nM β-mercaptoethanol. The plate was then heated to 75 °C for 10 min and the sample buffer containing the immune complexes that were eluted from the beads was transferred to 96-well PCR plates, sealed and stored at −20 °C until immunoblotting analysis.

### Mini-FLIP

Lysates from cells expressing the YFP-protein of interest were aliquoted into PCR tubes (100–120 μl per tube). FLIP was then conducted using the large volume FLIP procedure as described above, except 10 μl of protein A/G slurry per tube and 100 μl of Triton buffer for each wash was used. 10 μl of beads was collected during the last wash, and the fluorescence coating the beads was analyzed with a fluorescence microscope.

### Image Analysis

After IP, a distinct and sharp fluorescent ring around the edges of the beads was indicative of a positive FLIP, whereas a uniform, unfocused fluorescence on the bead was indicative of background signal. A simple examination of the collected pictures by eye is sufficient for qualitative determination of FLIP results, and discrimination of FLIP-positive from FLIP-negative antibodies.

In this report, FLIP analysis was performed using pictures of YFP-coated beads analyzed with ImageJ (Micro-manager 1.4.18, University of California). For a simple quantification of the total mean fluorescence performed using ImageJ, it was important that the beads were allowed to settle and form a uniform layer on the bottom of the plate, and that a comparable number of beads were analyzed for different IPs from the same lysate. A low but comparable number of beads was preferable over an excess of beads distributed in different layers in the well. An EVOS FL auto (Life technologies) or a BD-pathway microscope (BD bioscience) was used to automatically collect images of the YFP coating the beads after IP, but any fluorescence microscope with an appropriate filter should be suitable for the acquisition of FLIP images. 384-well black plates with clear bottoms were used and pictures were taken with a 20X magnification, keeping the microscope parameters (exposure time, contrast and gain) constant for all the pictures taken for the same experiment. All the pictures were then opened with ImageJ software and the brightness/contrast set at equal values for all the pictures of the same experiment. The total mean fluorescence of the pictures was then measured and the value obtained from the corresponding IgG control IP subtracted from the mean fluorescence obtained from the IP using the tested mAb (or FLAG Ab) ([mean fluorescence from IP using FLAG antibodies] – [mean fluorescence from IP using IgG control antibodies]).

### Software for FLIP Image Analysis

The FLIP image analysis software was developed in Python utilizing the Sci-Kit Image analysis package and a multitude of other supporting packages. The function of the software is to isolate and quantify the image signal from two regions of interest, the prominent bright ring at the perimeter of the agarose bead, and the body of the bead itself. Once the features are isolated, the average intensities of the respective regions are calculated and the FLIP signal is returned as the difference between the signals for the two regions. The true FLIP signal for the real-IP image was then calculated by subtracting the FLIP signal for its corresponding IgG control. Before using this software it is recommended that a uniform distribution of beads in the sample be visually confirmed in advance. A free and publicly available analysis tool for FLIP pictures can be found at http://openslice.fenyolab.org/flip.

## Results

### FLIP Assay Using HuEV-A Expression Vector

**Fig. 1:**
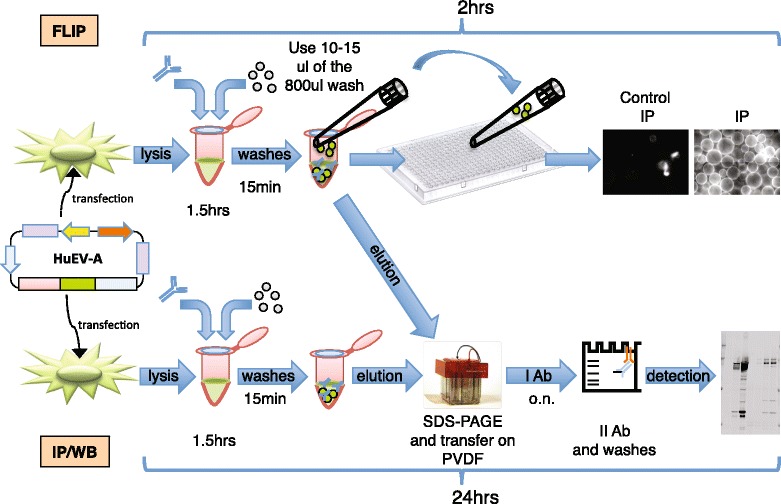
Comparison between FLIP and conventional IP/IB assay. The arrow connecting the two procedures shows how FLIP does not exclude the possibility of a subsequent conventional IP/IB analysis using the same starting material

**Fig. 2:**
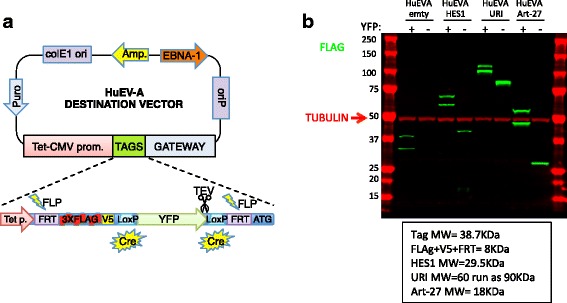
**a** Schematic of HuEV-A expression. ColE1 ori = bacterial origin of replication; Amp. = Ampicillin resistance cassette; EBNA-1 = Epstein-Barr nuclear antigen 1; OriP = origin of plasmid replication; Gateway = Gateway cloning cassette; Tet-CMV prom. = Doxycycline inducible promoter; FRT = flippase recognition target; FLP = flippase recombinase; LoxP = Lox sequence; Cre = Cre recombinase; TEV = TEV protease cleavage site. Note that treatment with Cre recombinase or FLP recombinase can produce a “short tag” or untagged derivative of the originally cloned ORF, respectively. **b** Immunoblot of cell lysates from HeLa cells expressing and empty HuEV-A vector or HES1, URI, or Art-27 proteins expressed from the HuEV-A expression vector. A version of the HuEV-A expression vectors not containing YFP in the tag (after Cre treatment of the vector) was also used for each of the proteins and for the empty vector. The proteins were detected with a FLAG-M2 antibody shown in green. Tubulin (shown in red) was used as loading control

Immunoprecipitation assays take advantage of the binding in solution of an antibody to a specific target peptide, protein or protein complex. Beads conjugated to protein A (for rabbit antibodies), protein G (for mouse antibodies) or to protein A and G (A/G), or to certain bacterial cells displaying these proteins on their surface, will bind the Ab-target complex, allowing the highly specific isolation of the target protein from a complex solution. The specificity is ensured by the highly selective interaction of the Ab to the target protein of interest. Washes of beads coated with the Ab-target complex ensure the clean purification/concentration/isolation of the target protein of interest. In standard IP/IB assays (immunoprecipitation followed by immunoblotting analysis) the target protein or complex of interest is eluted from the beads and subsequently visualized and analyzed by SDS-PAGE (SDS polyacrylamide gel electrophoresis) followed by immunoblotting. In contrast, in the FLIP assay the efficiency of IP is measured directly after IP, circumventing the need to run time-consuming and low-throughput gel electrophoresis and immunoblots (Fig. 1). This is made possible by expressing the target protein fused to a fluorochrome, for instance YFP (yellow fluorescent protein) as used here, but other fluorochromes can be chosen. To this end we developed a Gateway-compatible [18] human expression vector (HuEV-A) that adds a N-terminus tag to the protein of interest when the coding sequence is cloned into the HuEV-A vector. The tag encodes 3XFLAG, V5 and YFP (yellow fluorescence protein, Venus) [19] as tags. The coding DNA of interest can be easily cloned into the HuEV-A vector through the quick and highly efficient Gateway cloning system (Life Technologies, Prod. No 11791). The length of the tag added to the target protein can be shrunk as desired with FLIP or CRE recombinases to express an untagged protein, a 3XFLAG-V5 tagged protein or a 3XFLAG, V5 and YFP-tagged protein (Fig. 2a). In principle, any other vector enabling the tagging of a target protein of interest to a fluorochrome at either the N- or C-terminus, or even tagged internally, may be used.

**Fig. 3:**
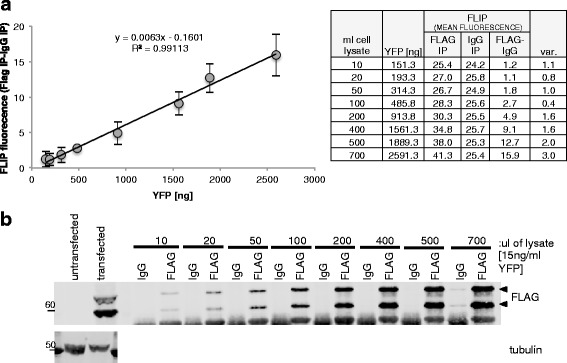
Comparison of FLIP and IP/IB sensitivity. **a** Correlation between amount of YFP used and FLIP signal. Two FLIP measurements from 2 different aliquots of beads were used to calculate the range of variability of the measurement (var.). **b** Immunoblot analysis of the same samples used in (**a**) A FLAG antibody was used as a positive control for IP and Western detection. Tubulin was used as a loading control for the total lysate used for IP and for the lysate of untransfected cells used as negative

**Fig. 4:**
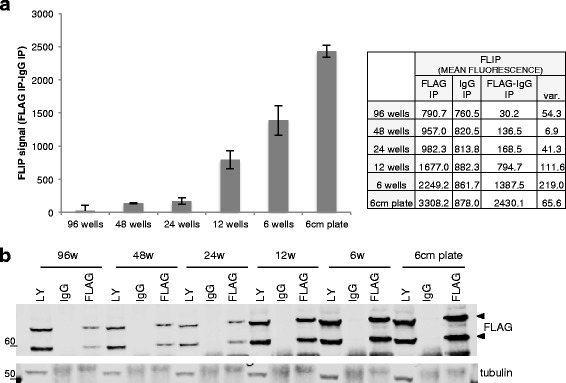
Evaluation of the amount of cells necessary for reliable FLIP. HeLa cells were plated, transfected and lysed in wells of different sizes. Lysates from each well were used to perform FLIP and IP/IB. **a** FLIP signal (total mean fluorescence measured by ImageJ) using a FLAG Ab subtracted from the FLIP signal using control IgG antibodies (background) is reported. Two FLIP measurements from 2 different aliquots of beads were used to calculate the range of variability of the measurement (var.). **b** The immunoprecipitated beads not used for FLIP were analyzed by immunoblotting. LY = input lysate; IgG = control IP using IgG antibodies; FLAG = IP of the tagged protein using anti FLAG antibodies. Tubulin was used as loading control

We have performed IP/IB analysis with hundreds of antibodies to identify those that are IP-grade. To facilitate this screening we cloned hundreds of protein coding regions into the HuEV-A vector. Proteins expressed as 3XFLAG, V5 and YFP fusion proteins consistently appear as double bands after SDS-PAGE and immunoblotting (Figs. 2b, 3, 4 and Additional file 1: Figure S4). The two bands migrate about ~10 kDa apart and are therefore more easily observable/sizable for smaller proteins. The two bands are derived from some unknown property of the overexpressed YFP. We performed mass spectrometry analysis of the proteins isolated from the upper and lower bands of two randomly chosen proteins (HES-1 and URI) expressed from the HuEV-A vector. In both analyses, the nature and quantity of the observed peptides from the upper and the lower bands were indistinguishable (Additional file 1: Figure S1 and S2), suggesting that both bands contained full-length proteins with a complete tag. To narrow down the region of the tag responsible for the double band we performed CRE-mediated recombination using HuEV-A vectors expressing HES-1, URI and Art-27 proteins. Three “daughter” expression vectors lacking the YFP in the tag were therefore obtained. Expression in HeLa cells of the 3XFLAG, V5 (8KDa) tagged proteins and the 3xFLAG, V5, YFP (38KDa) tagged proteins showed that the double band becomes a single band of expected size when the YFP is absent (Fig. 2b, even lanes). The two bands are therefore derived from some unknown property of the overexpressed YFP such as two alternate folded states. Further investigation is being conducted to elucidate this phenomenon. For the purpose of development and characterization of the FLIP assay, the presence of a double band after IP/WB was not relevant and therefore we utilized the HuEV-A vector for all the following analyses.

In the FLIP assay, following cell lysis and incubation of lysate, beads, and antibody, the bead/immune-complex mixture is washed and then directly visualized with a fluorescence microscope or automated microscopy system. If the YFP-tagged protein of interest has been successfully immunoprecipitated the beads will be coated with a fluorescent signal detectable under light with an appropriate excitation wavelength. On the other hand, if the IP fails, the beads will not fluoresce and no signal will be detected by fluorescence microscopy. Quantification of the fluorescence signal of the beads against a control signal from a control IP performed using immunoglobulins from a non-immunized mouse (IgG) provides a reliable indication of the success of the IP (Fig. 1).

In contrast to the standard IP/IB analysis, the FLIP assay is a much faster procedure because it is based on the direct observation of fluorescent target proteins coating the beads. Moreover the FLIP assay can be integrated into any IP workflow since FLIP analysis requires only a small fraction of the bead/immune-complex product; the left-over beads can be processed for elution and follow-up analysis as required (Fig. 1).

### FLIP is a Sensitive and Scalable Assay

Next we characterized the sensitivity of the FLIP assay compared to standard IP/IB procedures. For these analyses we used proteins overexpressed from the HuEV-A vector that, despite the still double band visible by immunoblotting analysis, is an easy to clone vector that enables Tet-regulation and high expression in mammalian cells.

To interrogate the efficiency and sensitivity of the FLIP assay compared to standard IP/IB, a randomly chosen protein (HES-1) expressed from the HuEV-A vector was used together with a well-known commercial antibody against FLAG-tag (Anti-FLAG M2; Biorad). Different amounts of the HuEV-A expressed protein/cell lysate were used to perform FLIP and IP/IB in parallel. The tagged-HES-1 was expressed in previously transfected Tet-on HeLa cells (see experimental procedures). The fluorescence of the cell lysate was measured and the amount of YFP per microliter of solution deduced using a standard curve of purified YFP (Additional file 1: Figure S3). Different amounts of cell lysate derived from cells expressing the tagged protein were used in both FLIP assays or standard IP/IB using an anti-FLAG commercial antibody (Fig. 3a, b). The FLIP signal calculated using ImageJ was plotted versus the corresponding amount of YFP-protein used for the assay (Fig. 3a). We observed a linear increase of fluorescent signal over background by FLIP similar to the increase of the Immunoblot signal following IP, indicating a very similar sensitivity between the FLIP assay and the IP/IB assay.

To identify the minimum amount of cells necessary to obtain a reliable FLIP signal, the lysates from HeLa cells transfected and cultured in different size plates (96, 48, 24, 12, 6-well plates or 6-cm plates) were used to perform FLIP and IP/IB analysis in parallel. Even with minimal starting material (0.02×10^6^ cells plated in one well of a 96-well plate) we observed a low but positive FLIP signal. For all the utilized conditions, a good correlation between the FLIP and the IP/IB analyses was observed (Fig. 4). A reliable FLIP signal consistently above background signal was obtained from 48-well plates.

Overall these observations show an excellent correlation between standard IP/IB analysis as compared to FLIP analysis. Therefore, the FLIP assay presents sensitivity comparable to the classical IP/IB analysis while minimizing assay time and sample handling. These features confer to the assay a much higher potential for high-throughput. These advantages of FLIP make our new technique a relevant and appealing tool for several molecular biology applications including the high-throughput screening of antibodies of unknown performance in immunoprecipitation.

### High-Throughout FLIP Screening for IP Competent Mouse Antibodies

**Fig. 5:**
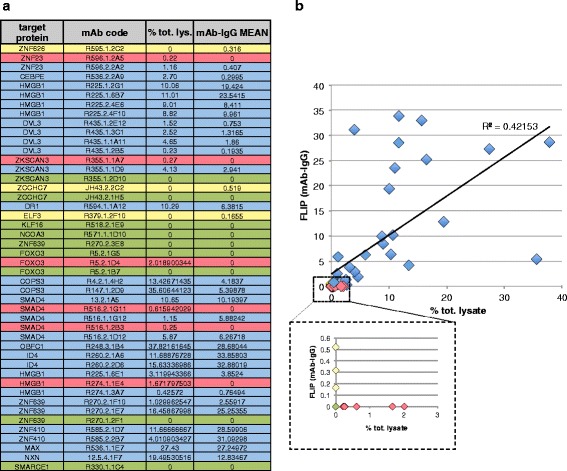
High-throughput FLIP applied to the screening of IP-grade mAbs. **a** 46 mAb produced and purified by CDI laboratories were tested by FLIP and immunoblotting (Additional file 1: Figure S3). The FLIP signal (mAb FLIP subtracted from the control IgG FLIP) and the % of the immunoprecipitated target protein measured after immunoblotting (% tot. lys.) are reported together with the name of the target proteins and the code specific for each antibody. Negative FLIP values (for which the FLIP signal of the background IP is greater than the FLIP signal after IP with the considered mAb) are reported as zero. **b** The FLIP values calculated using ImageJ and the % tot. lysate index obtained after immunoblotting are plotted and the R^2^ of the best fit trend-line is reported. The inset shows the plotted values for mAbs that had a positive FLIP value but did not show a band for the immunoprecipitated target protein by western analysis (FLIP false positives in yellow), mAbs that were shown not to be IP-competent by both FLIP and IP/western (in green), and mAbs that did not have a positive FLIP value but showed a band for the immunoprecipitated target protein by immunoblot analysis (FLIP false negatives in red). In blue are reported all the mAbs that show good accordance between FLIP and IP/western analysis

To directly test the application of FLIP in the high-throughput identification of IP-grade antibodies we performed FLIP on 46 different mouse monoclonal antibodies (mAb) produced by CDI Laboratories, targeting 23 different proteins. The antibodies were tested using a 96-well plate IP procedure described in Methods. A 10 μl aliquot of the immunoprecipitated beads (about 1.25 % of the beads used for IP) were collected during the last wash and used for FLIP analysis; the rest of the beads (98.75 %) were used for IP/IB analysis as depicted in Fig. 1. The efficiency of a specific mAb in precipitating the target protein expressed in HeLa-M2 cells from a HuEV-A expression vector was quantified after Immunoblotting (Additional file 1: Figure S5), comparing the amount of the target protein in the total lysate with the amount of immunoprecipitated protein. This calculated index (% of total lysate) was correlated with the FLIP signal calculated using ImageJ (mean fluorescence of the beads after IP, with the analyzed mAb subtracted from the mean fluorescence after IP with control mouse IgG antibodies). The results of this analysis (Fig. 5) showed a good correlation between the FLIP analysis and the IP/IB analysis. The majority of the antibodies positive for FLIP were also positive for IP/IB analysis (28/34) and most of the antibodies that failed IP/IB also failed FLIP (9/12). This equal to a success rate of about 80 % (37/46 Abs, as shown in Fig. 5). Out of the 46 mAbs tested, 3 (6.5 %) were FLIP false positives (did not pass IP/IB but had a positive FLIP, shown in yellow in Fig. 5); unlike the true positives, 23/28 of which had FLIP signals >1.0, all of the false positive antibodies had FLIP signals <0.6. Also, 6 antibodies (13 %) were FLIP false negative (negative for FLIP but positive for IP/IB, shown in red in Fig. 5). These 6 FLIP false negative antibodies had a % IP index <2, consistent with Abs with relatively low affinity for their target proteins.

Overall these observations demonstrate the high-throughput capability of the FLIP assay applied for the screening of antibodies able to efficiently immunoprecipitate their target protein.

### High-Throughput Mini-FLIP

**Fig. 6:**
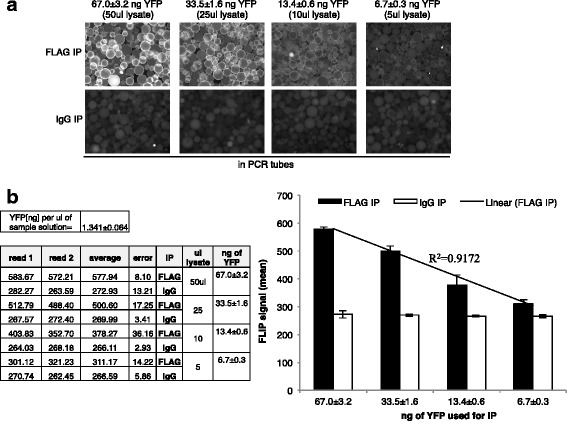
Mini FLIP. **a** Decreasing amounts of a YFP-tagged protein (HES-1) expressed in HeLa cells transfected with a HuEV-A construct were used for FLIP analysis in just 100 ml of total volume (images show one of the two pictures used for the analysis). IgG antibodies were used as for previous experiments as control, while FLAG antibodies were used to IP the YFP-tagged target protein. **b** Comparison of the FLIP signal obtained using control IgG antibodies and FLAG antibodies that specifically IP the target protein shows a FLIP signal higher than background, even when using the lower amount of YFP-protein (6.7 ng YFP/5 ml of lysate). The values reported in the tables are graphed in the histogram. The linear correlation R^2^ value between the amount of YFP used for IP and the FLIP signal is reported

To improve the throughput capability, we performed FLIP in 0.2 mL PCR tubes using a total volume of just 100 μl (mini-FLIP). Different amounts of lysates were tested to identify the minimum amount of YFP-protein necessary to obtain a reliable FLIP signal. HeLa-M2 cells transfected with a HES-1-expressing HuEV-A construct were also used for this analysis. Different amounts of lysate (containing 1.34 ± 0.064 ng YFP per μl as deduced by YFP fluorescence of the solution) were used to perform FLIP using a FLAG M2 (Biorad) antibody and a total volume of 100 μl. Here we show a positive FLIP (signal obtained from FLAG IP higher than the signal obtained from IgG control IP, which was considered as background) using just 6.7 ± 0.3 ng of YFP (5 μl of lysate) and a signal considerably higher than background using 13.4 ± 0.6 ng of YFP (10 μl of lysate) (Fig. 6). This result shows that “miniaturization” of the FLIP assay is readily achieved for high-throughput screening of antibodies.

### FLIP can be Used to Screen Unpurified Antibodies

**Fig. 7:**
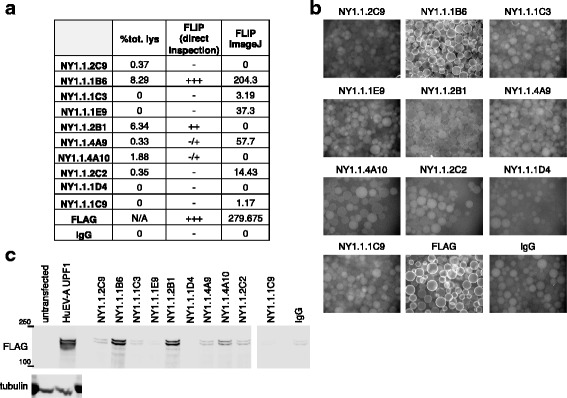
FLIP applied to hybridoma supernatants. 10 hybridoma supernatants produced after immunization with a UPF1 polypeptide were tested by FLIP and IP/IB. **a** The results of IP/IB, direct examination of the pictures collected after FLIP and ImageJ analysis of the FLIP results are reported in the table. **b** One of the two pictures collected after FLIP is also shown for each of the supernatants, FLAG and IgG IP. **c** IP/IB analysis of the 10 supernatants tested. Immunoblotting was performed using a FLAG antibody and tubulin was used as loading control

**Fig. 8:**
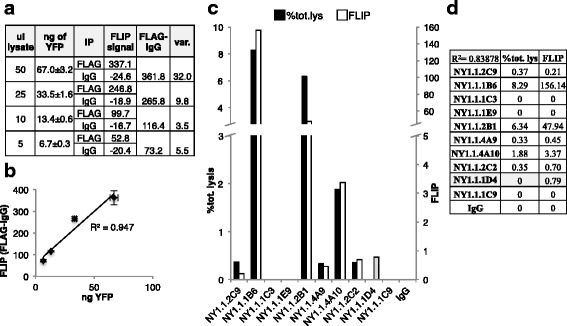
Development of software for the automated and improved analysis of FLIP images. **a**-**b** A new method to quantify FLIP signals described in the text and in “Experimental procedures” was applied to the FLIP analysis presented in Fig. 5 (mini-FLIP). The values reported in the table (**a**) are graphed in the scatter plot (**b**) and the R^2^ of the best-fit trend line is reported. The range of variability (var.) is calculated from 2 measurements of the fluorescence of the input lysate used for FLIP (X axis) and from 2 separate measurements of the FLIP signal from the beads (Y axis). **c**-**d** The improved FLIP analysis was applied to the screening presented in Fig. 6. The % of total lysate immunoprecipitation index (% tot. lys) calculated after IP/IB, and the improved FLIP signal, are reported in the column graph and in the table. The only supernatant (NY1.1.1D4) showing a discrepancy between IP/IB and FLIP is highlighted in gray

If a quick functional screen could be performed before purification of antibodies from hybridoma supernatants, considerable amounts of time, material and money could be saved in avoiding purification and subsequent testing of antibodies that fail to perform or perform poorly in immunoprecipitation. The feasibility of using FLIP with unpurified antibodies was therefore tested. 10 hybridoma supernatants produced by CDI Laboratories were used to perform mini-FLIP and IP/IB (Fig. 7). The hybridomas were produced from mice immunized with a UPF-1 (target protein) polypeptide (416 a.a.) purified from an *E. coli* overexpression plasmid; lysates from HeLa-M2 cells transfected with a HuEV-A-UPF1 vector were used for IP/IB and FLIP testing. IP/IB analysis revealed two supernatants (NY1.1.1B6 and NY1.1.2B1) that worked very efficiently in IP, one supernatant (NY1.1.4A10) with a reasonable efficiency at recognizing and IPing its target protein, and three supernatants (NY1.1.2C9, NY1.1.4A9 and NY1.1.2C2) with very weak IP capability, but still presenting signals distinguishable from the background (IgG IP) (Fig. 7a, % tot. lysate column and 7c). ImageJ analysis of the fluorescence coating the beads after FLIP (Fig. 7a, FLIP ImageJ column) failed to reliably predict the performance of the tested supernatants in IP, and was able to identify only the highest performing hybridomas (NY1.1.1B6 and NY1.1.2B1). This was probably due to a high background fluorescence signal and the noticeable variation of bead number in the different wells during FLIP assay. Direct examination of the collected FLIP pictures also clearly showed a positive FLIP for the two best performing supernatants (NY1.1.1B6 and NY1.1.2B1), as judged by the clear fluorescent ring on the edges of the beads (Fig. 7b). Although direct visual evaluation of the pictures from FLIP was able to easily identify the supernatants with higher performance in IP, this approach also failed to clearly identify the supernatants with low IP performance. We therefore developed software for the more accurate analysis of FLIP images (Fig. 8). This improved FLIP analysis was able to almost perfectly predict IP behavior of all the supernatants tested (Fig. 8c, d). The only exception was NY1.1.1D4 that passed FLIP analysis but did not pass IP/IB evaluation. Correlation of the % of total lysate index and the improved FLIP signal showed a linear correlation with *R*^2^ = 0.83878, suggesting a good concordance between the IP/IB analysis and FLIP analyzed with our software (further described below).

This result demonstrated that unpurified antibodies can be efficiently used for FLIP analysis to predict the IP performance of the tested immunoglobulins.

### Development of Software for Automated Analysis of FLIP Images

The quantification of the FLIP signals presented in Figs. 3, 4, 5, 6 and 7 was done using ImageJ by measuring the total mean fluorescence of the acquired images (see Experimental Procedures). This analysis, although extremely simple and accessible to any laboratory, does not account for the number of beads as well as their area, and is extremely sensitive to possible fluorescent contaminants that aberrantly increase the measured FLIP signals. Moreover we observed that in control IPs using normal mouse IgG certain agarose beads exhibited high fluorescence. Although high, this background fluorescence was easily distinguishable from the fluorescence caused by specific IP because the background fluorescence was always uniformly distributed on the beads, whereas the IP signal was clearly characterized by a ring of strong fluorescence around the beads. We therefore developed software that measures and compares the fluorescence on the rims of the beads with the fluorescence present on the body of the beads. This procedure was applied to every bead in the picture taken as a single entity. Quantifying FLIP signals in this way therefore is independent of the bead number and excludes signals from non-spherical shapes (likely contaminants and/or overlapping beads). We applied the improved analysis method to the FLIP analysis presented in Fig. 6 (mini-FLIP) and Fig. 7 (FLIP using unpurified antibodies) and obtained a remarkable improvement in FLIP performance, especially when used for more challenging applications such as using unpurified antibodies (Fig. 8). The linear correlation (R^2^) between the amount of target protein used for IP and mini-FLIP signal was slightly improved using the new analysis (from 0.917 to 0.947) (Fig. 6c versus b). More importantly, we observed a great increase in separation between FLIP signal from FLAG IP or tested antibody IP and background signal (IgG IP) (respectively, Fig. 6b versus Fig. 8a and Fig. 7 versus Fig. 8c and d). The quantified FLIP signal from control IPs was always negative using the new methodology of analysis, reflecting the fact that the edges of the control beads were actually darker than the body of the beads (Fig. 8a).

Moreover, the improved analysis clearly outperformed the ImageJ analysis in FLIP done using unpurified antibodies (Figs. 7 and 8c, d). Analysis of FLIP results with the improved method produced FLIP values that almost perfectly correlated with the IP/IB in our screening of hybridoma supernatants produced after immunization of mice with UPF1 protein (Fig. 8c, d and previous paragraph).

The principles used by the new quantification method are described in further details in the “experimental procedures” section. A publicly available analysis tool for FLIP pictures can be found at http://openslice.fenyolab.org/flip.

This new tool improves the sensitivity of the FLIP, especially for the low performing antibodies and moreover increases the throughput of the FLIP assay, rendering this method an extremely easy, fast, and reliable way to screen antibodies or even hybridoma supernatants for applications in immunoprecipitation.

## Discussion

Immunoprecipitation (IP) procedures are applied to a large variety of molecular biology assays, including protein purification, concentration, co-immunoprecipitation and chromatin immunoprecipitation [2–8]. The classical IP procedure is often coupled to immunoblotting analysis, a technique suited to low throughput analysis of only a few samples [10, 20, 21]. Here we describe and characterize FLIP (Fluorescence IP), which couples conventional IP to the direct observation and/or quantification of a fluorescently tagged target protein on the surface of beads. This approach is faster and cheaper than using IB to measure the success of an IP, and is thus amenable to high-throughput screens. A major limitation of the IP assay is often the availability of antibodies able to efficiently immunoprecipitate the target protein [22]. Here we applied FLIP to the screening of mouse monoclonal antibodies in order to quickly screen and identify those able to immuneprecipitate their corresponding target protein.

Here we show that FLIP has a similar sensitivity to the IP/IB assay and that FLIP can also be miniaturized to increase throughput by minimizing both volume of cell lysate and amount of antibody used for IP. Another advantage of FLIP is the use of fluorescently labeled target protein, which ensures a degree of specificity for the antibodies that pass FLIP. Indeed, given that the target protein is the only fluorescently tagged protein in the cell lysate used for IP, the presence of fluorescence coating the agarose beads after immunoprecipitation indicates selective binding of the tested antibody to the target protein. On the other hand, the FLIP assay is “blind” to any possible non-specific binding of the tested antibody to proteins different from the fluorescently labeled target protein. Another possible limitation of FLIP is that it does not currently give any information about the ability of the tested antibody to recognize the target protein in its denatured state and therefore FLIP cannot predict the behavior of antibodies in immunoblotting assays. To this end, FLIP could theoretically be used using protein solutions/lysates pre-treated with denaturing conditions such as high SDS concentrations and/or reducing agents [5]. This approach may be possible and somehow informative because of the high stability of GFP and GFP derived fluorophores that can withstand denaturing conditions undergoing partial denaturation and still remaining fluorescent [23].

Also, FLIP cannot assay the performance of an antibody in IP of endogenous proteins because it relies on the overexpression of the target protein. Nevertheless, we show here that FLIP can easily be integrated into classical IP/IB procedures with no disruption and no diminished sensitivity of the latter. This allows for further characterization of the antibodies after FLIP screening for the IB analysis of bands of the endogenous protein. Moreover, FLIP relies on the overexpression of the target protein as a fluorophore-tagged fusion and therefore requires the cloning of the gene of interest into a suitable expression vector. To this end we developed a flexible mammalian expression vector named HuEV-A that combines the simplicity of Gateway cloning with the flexibility of our 3xFLAG-V5-YFP tag [18, 19, 24]. We used the Life Technologies Ultimate ORF collection, compatible with Gateway cloning (Gateway entry clone format) to construct a HuEV-A library of proteins. Also, the HuEV-A tag can be dramatically reduced in size with simple Cre recombination to a 3xFLAG-V5 tag, or the entire tag can be eliminated by employing FLP recombination.

The FLIP assay is very similar in basic concept to the previously described LUMIER (luminescence-based mammalian interactome mapping) assay [12, 13] which has been applied to a wide range of applications, including identification of new protein-protein interactions [12, 25–27], validation of yeast two hybrid screening hits [28, 29] and identification of particular antibodies in patients’ blood (Luciferase Immunoprecipitation Systems or LIP) [30]. LUMIER exploits the overexpression of a target protein fused to Renilla luciferase and utilizes quantification of luminescence signal after immunoprecipitation. As for FLIP, the success of the immunoprecipitation of the target fusion protein can be efficiently and easily determined by high-throughput measurements: measurement of luminescence after incubation with a luciferase substrate in the case of LUMIER and LIP, and measurement of the fluorescence coating the immunoprecipitated beads in the case of FLIP. Despite the great success and the many applications of the LUMIER system no alternative assays with high-throughput capability has been developed for IP analysis until now.

Our FLIP assay is not meant to substitute for the LUMIER assay, which is probably more sensitive compared to IP/WB and FLIP analysis itself. Our FLIP assay is meant to complement the previously known techniques (IP/WB and LUMIER assays) in high-throughput procedures challenging for luciferase reactions or in contexts in which microscopy observations are more amenable than luciferase signal quantification or in which fluorophore tagging is preferable to Renilla tagging. The FLIP assay is an additional tool for the fast screening of IP reactions. FLIP can also be envisioned as an orthogonal assay to the LUMIER system because “preys” tagged with FLAG and a fluorophore of choice can be used with Renilla tagged “baits” (see [12]) to verify and quantify efficiency of IP before luminescence quantification. In this context our HuEV-A expression vector that includes a FLAG, V5 and YFP tag would be optimal.

Compared to IP-Western blotting, some information such as the nonspecific reactivity of the antibody towards non-target proteins, and/or the preferential reactivity towards specific isoforms or splice variants of the target protein, are lost. Nevertheless, we foresee an extremely useful application of FLIP in screening for antibodies that function efficiently in IP assays. For instance, many unpurified antibodies can be tested by FLIP directly using hybridoma supernatants. After selection of the FLIP-positive clones, a reduced number of the antibodies would need to be purified and characterized further by IP/IB or other assays. Moreover, because of the minimal material used in the FLIP analysis, a new IP does not need to be performed again for the FLIP-positive antibodies, as the left-over beads from FLIP analysis can be used for the follow-up characterizations.

Here we show that FLIP is a fast and reliable method that can partially substitute and easily complement the conventional IP/IB procedure. This new technique can be directly applicable to the high-throughput screening for the identification of IP-grade antibodies.

## Conclusions

Immunoprecipitation (IP) is a powerful and informative procedure often coupled to immuno-blotting analysis (IP/IB). IP/IB has limited scalability to high-throughput pipelines. Here we describe a novel, fast and easily scalable method called FLIP (FLuorescence IP) that can partially substitute and complement the conventional IP/IB procedure. Beside the description and application of the new method, we also built and characterized a flexible plasmid (HuEV-A) for YFP protein tagging and we developed a publicly available analysis tool for FLIP pictures that improves the sensitivity of FLIP.

## Abbreviations

Ab, antibody; FLIP, fluorescence-IP; IB, immunoblot; IgG, immunoglobulin; IP, immunoprecipitation; IP/IB, immunoprecipitation followed by immunoblot analysis; mAb, mouse monoclonal antibody; S.D., standard deviation

## Additional file

Additional file 1: Figure S1. Mass spectrometry of tryptic peptides of YFP fusion proteins recovered from upper and lower bands of HES-1 expressed the HuEV-A vector. **Figure S2.** Mass spectrometry of tryptic peptides of YFP fusion proteins recovered from upper and lower bands of URI as in **Figure S1.** **Figure S3.** Standard curve of YFP amounts versus fluorescence reading (excitation 475 nm, emission 527 nm). **Figure S4.** a) HeLa cells were plated and transfected with Fugene-HD (Promega) in different size wells according with the parameters reported in the table. b) After lysis of the cells in each of the wells, the YFP fluorescence of 10 ml of solution used for IP was 2 measured. **Figure S5.** Tet-ON HeLa cells were transfected with the HuEV-A construct encoding the target protein with an N-terminal FLAG, YFP (Venus) and V5 tag under a tet-inducible promoter. (PDF 5435 kb)
