# Supplementary material for: Fluorescence ImmunoPrecipitation (FLIP): a Novel Assay for High-Throughput IP
Source: Biol Proced Online. 2016 Aug 15;18:16. doi: 10.1186/s12575-016-0046-x (PMC4983793; doi:10.1186/s12575-016-0046-x)
Supplement: Additional file 1: Figure S1. — Mass spectrometry of tryptic peptides of YFP fusion proteins recovered from upper and lower bands of HES-1 expressed the HuEV-A vector. Figure S2. Mass spectrometry of tryptic peptides of YFP fusion proteins recovered from upper and lower bands of URI as in Figure S1. Figure S3. Standard curve of YFP amounts versus fluorescence reading (excitation 475 nm, emission 527 nm). Figure S4. a) HeLa cells were plated and transfected with Fugene-HD (Promega) in different size wells according with the parameters reported in the table. b) After lysis of the cells in each of the wells, the YFP fluorescence of 10 ml of solution used for IP was 2 measured. Figure S5. Tet-ON HeLa cells were transfected with the HuEV-A construct encoding the target protein with an N-terminal FLAG, YFP (Venus) and V5 tag under a tet-inducible promoter. (PDF 5435 kb) [file 12575_2016_46_MOESM1_ESM.pdf]

Supplemental figure legends

Figure S1. Mass spectrometry of tryptic peptides of YFP fusion proteins recovered from upper and lower bands of HES-1 expressed the HuEV-A vector. Coverage maps were prepared using Byonic software. Shown is the sequence of HES1 tag and N terminus. Amino acids denoted in green are peptides identified in this analysis. Each green line under a peptide denotes the number of times the peptide has been identified in this analysis (spectral counts). A red line below a peptide indicates that the corresponding amino acid carries a modification (in vivo or in vitro). For instance, cysteines are reduced and alkylated with iodoacetamide prior to analysis and are therefore all underlined in red. The red arrow indicates the beginning of the protein and end of the tag.

Figure S2. Mass spectrometry of tryptic peptides of YFP fusion proteins recovered from upper and lower bands of URI as in figure S1. Shown in a) is the sequence of the URI tag as in S1. URI protein spectral counts downstream of the tag are shown in b).

Figure S3. Standard curve of YFP amounts versus fluorescence reading (excitation 475nm, emission 527nm). The R^2 and equation of the best-fit trend-line for the experimental points is also reported.

Figure S4. a) HeLa cells were plated and transfected with Fugene-HD (Promega) in different size wells according with the parameters reported in the table. b) After lysis of the cells in each of the wells, the YFP fluorescence of 10 ml of solution used for IP was

measured. The range of variability between two separate FLIP measurements (var.) is reported.

Figure S5. Tet-ON HeLa cells were transfected with the HuEV-A construct encoding the target protein with an N-terminal FLAG, YFP (Venus) and V5 tag under a tet-inducible promoter. These cells were stimulated with 0, 50 or 1000 ng/ml doxycycline. Immunoprecipitation (IP) was carried out using 5 μg of either IgG, tested CDI mAb, or 5 μg of FLAG-M2 (Sigma). Immunoblotting was performed using rabbit Anti-FLAG (1:1000 dilution, Cell Signaling #2368). The arrow indicates the predicted molecular weight of the target protein.

Figure S1

HES1 _ TOP _Band

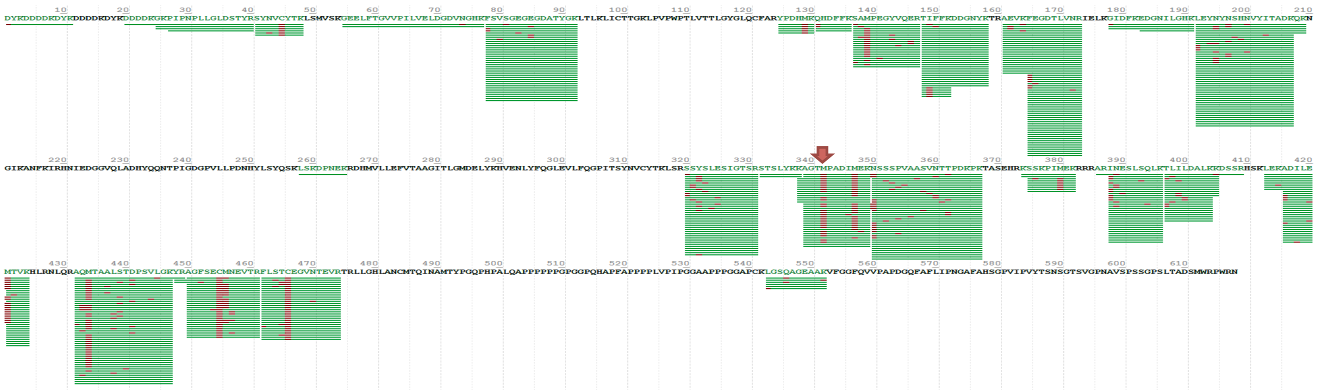

HES1 _ BOTTOM_band

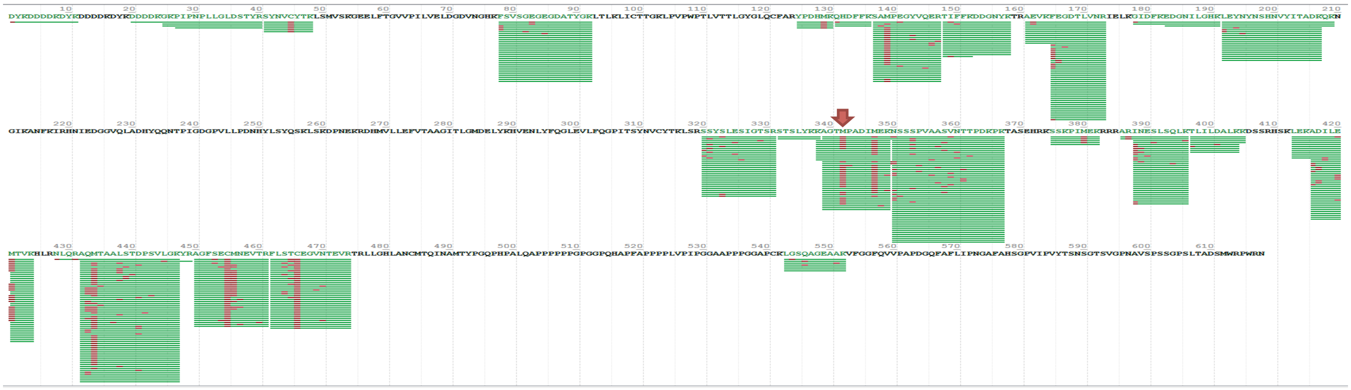

Figure S2 a)

URI _ TOP _Band_Tag_sequence_Portion

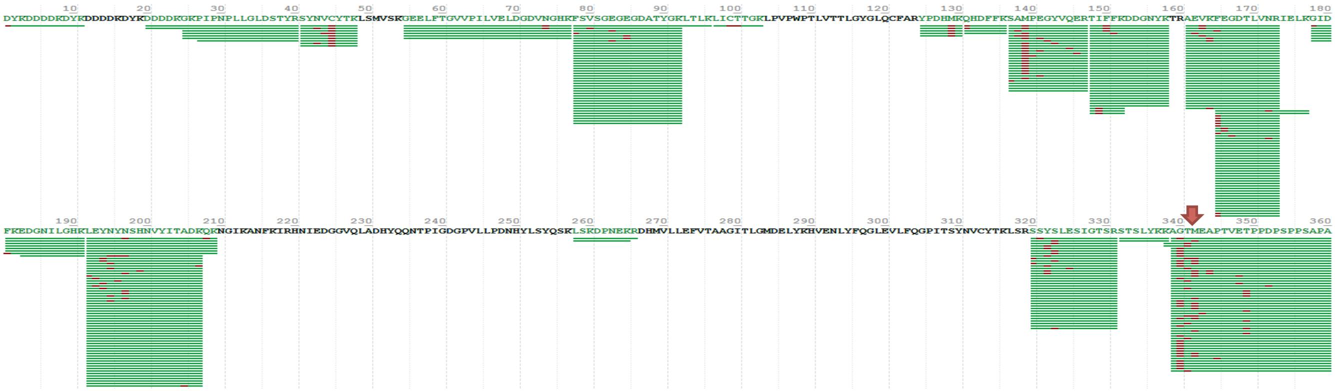

URI _ BOTTOM_ Band_Tag_sequence_Portion

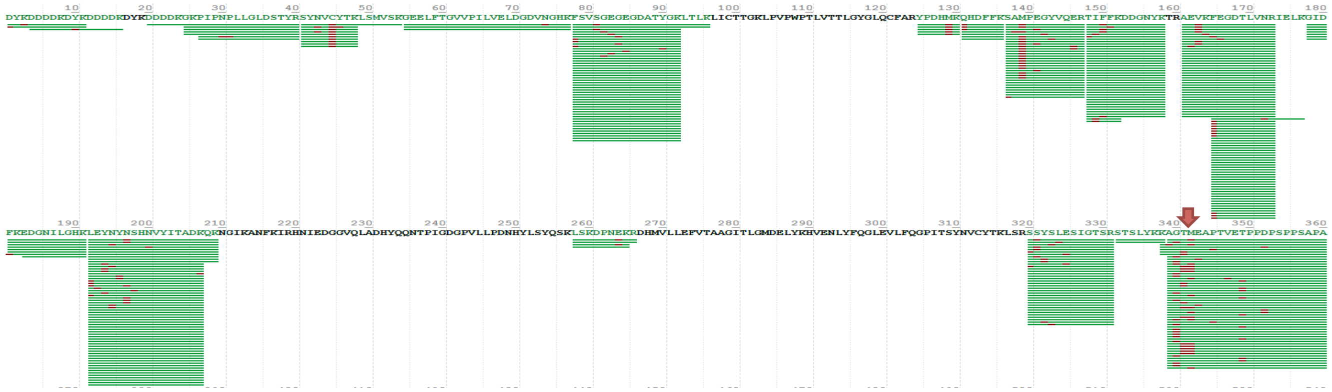

Figure S2 b)

URI _ Top_Band_Protein_sequence_Portion

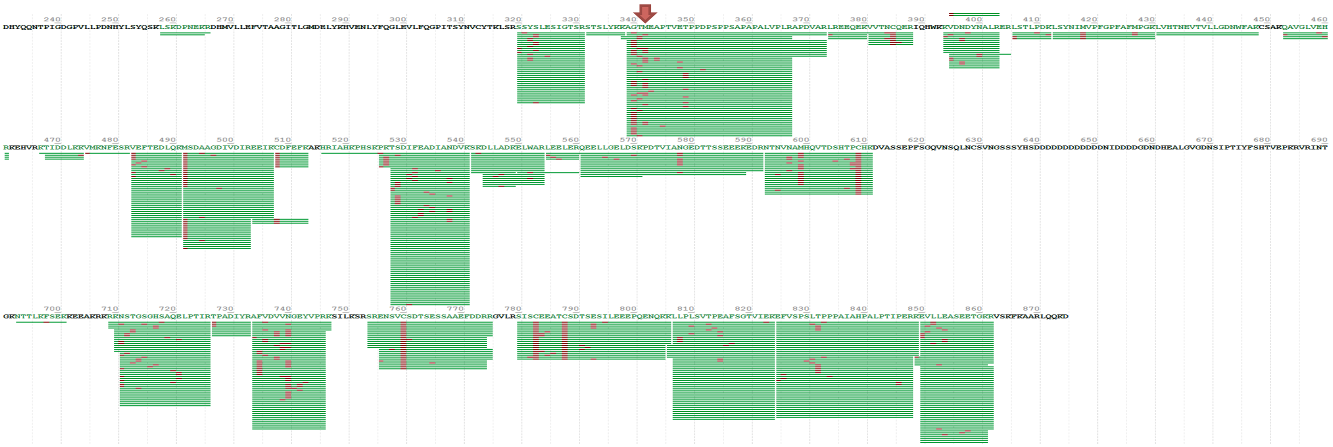

URI _ Bottom _Band_Protein_sequence_Portion

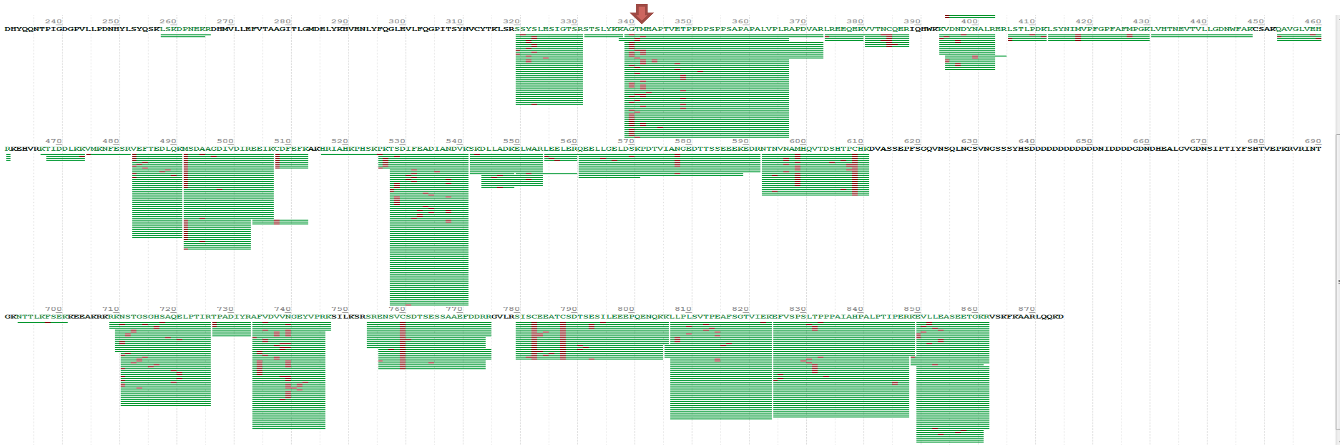

Figure S3

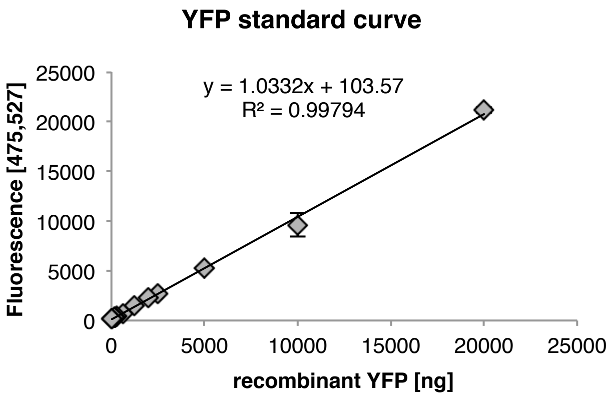

YFP standard curve
y = 1.0332x + 103.57
R² = 0.99794
Fluorescence [475,527]
25000
20000
15000
10000
5000
0
0
5000
10000
15000
20000
25000
recombinant YFP [ng]

Figure S4

A

	cells/well [X10^6]	media [ul]	DNA [ug]	OPTIMEM [uL]	Fugene-HD
96 well/plate	0.02	100	0.075	10	0.225
48 well/plate	0.03	200	0.1	10	0.4
24 well/plate	0.06	400	0.2	25	0.8
12 well/plate	0.12	800	0.4	25	1.5
6 well/plate	0.3	2000	0.75	50	3
6cm plates	0.6	2500	1.5	100	6

B

SAMPLES USED FOR IP	YFP fluorescence	var.
94 wells	22.5	8.9
48 wells	51.5	17.4
24 wells	52	5.4
12 wells	282	30.8
6 wells	819.5	18.8
6cm plate	1434.5	76.8

Figure S5-1

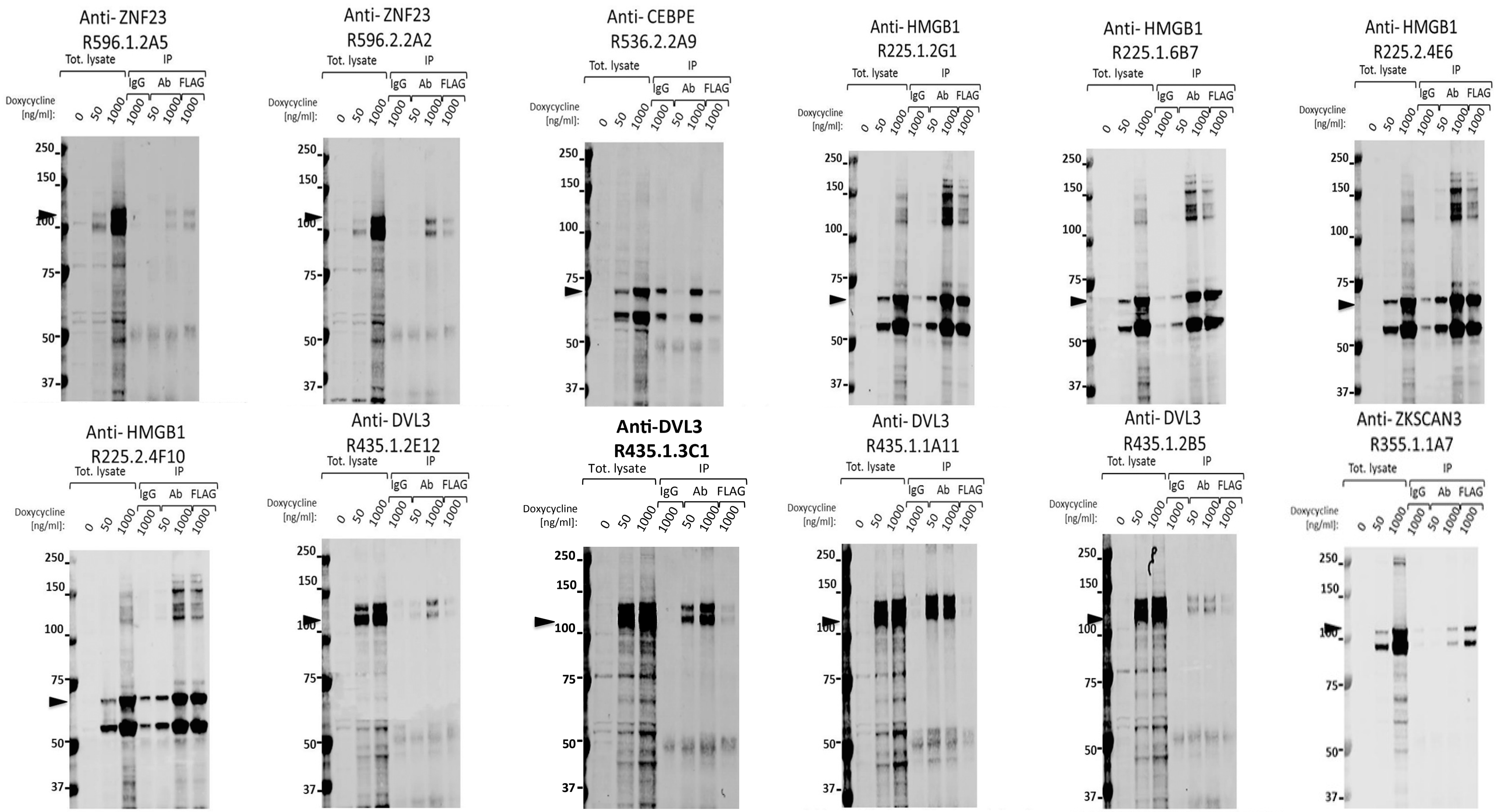

Anti- ZNF23
R596.1.2A5
Anti- ZNF23
R596.2.2A2
Anti- CEBPE
R536.2.2A9
Anti- HMGB1
R225.1.2G1
Anti- HMGB1
R225.1.6B7
Anti- HMGB1
R225.2.4E6
Anti- HMGB1
R225.2.4F10
Anti- DVL3
R435.1.2E12
Anti-DVL3
R435.1.3C1
Anti- DVL3
R435.1.1A11
Anti- DVL3
R435.1.2B5
Anti- ZKSCAN3
R355.1.1A7
Tot. lysate
IP
IgG
Ab
FLAG
Doxycycline [ng/ml]:
0
50
1000
250
150
100
75
50
37

Figure S5-2

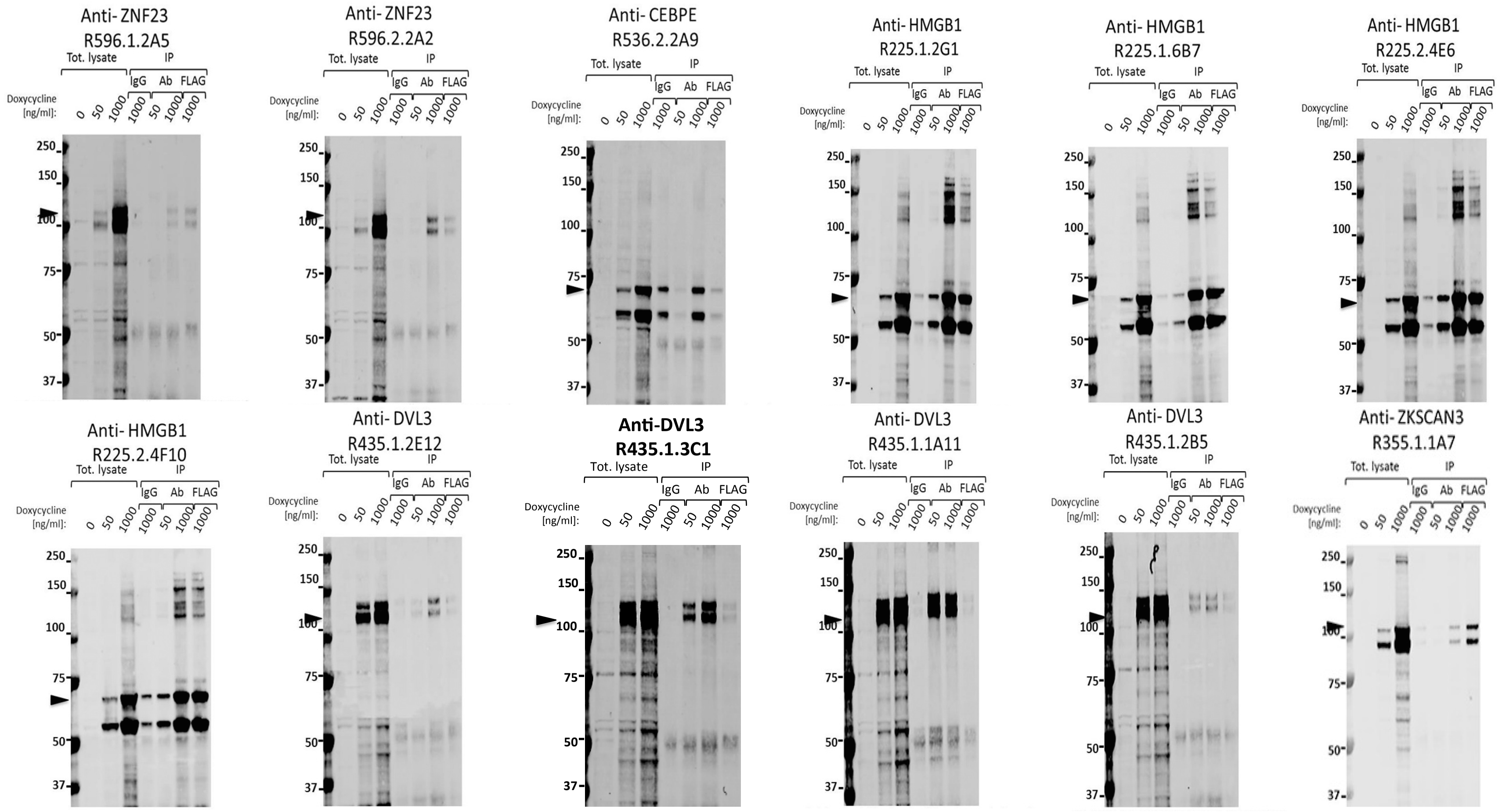
Anti- ZNF23
R596.1.2A5
Anti- ZNF23
R596.2.2A2
Anti- CEBPE
R536.2.2A9
Anti- HMGB1
R225.1.2G1
Anti- HMGB1
R225.1.6B7
Anti- HMGB1
R225.2.4E6
Anti- HMGB1
R225.2.4F10
Anti- DVL3
R435.1.2E12
Anti-DVL3
R435.1.3C1
Anti- DVL3
R435.1.1A11
Anti- DVL3
R435.1.2B5
Anti- ZKSCAN3
R355.1.1A7
Tot. lysate
IP
IgG
Ab
FLAG
Doxycycline [ng/ml]:
0
50
1000
1000
50
1000
1000
250
150
100
75
50
37

Figure S5-3

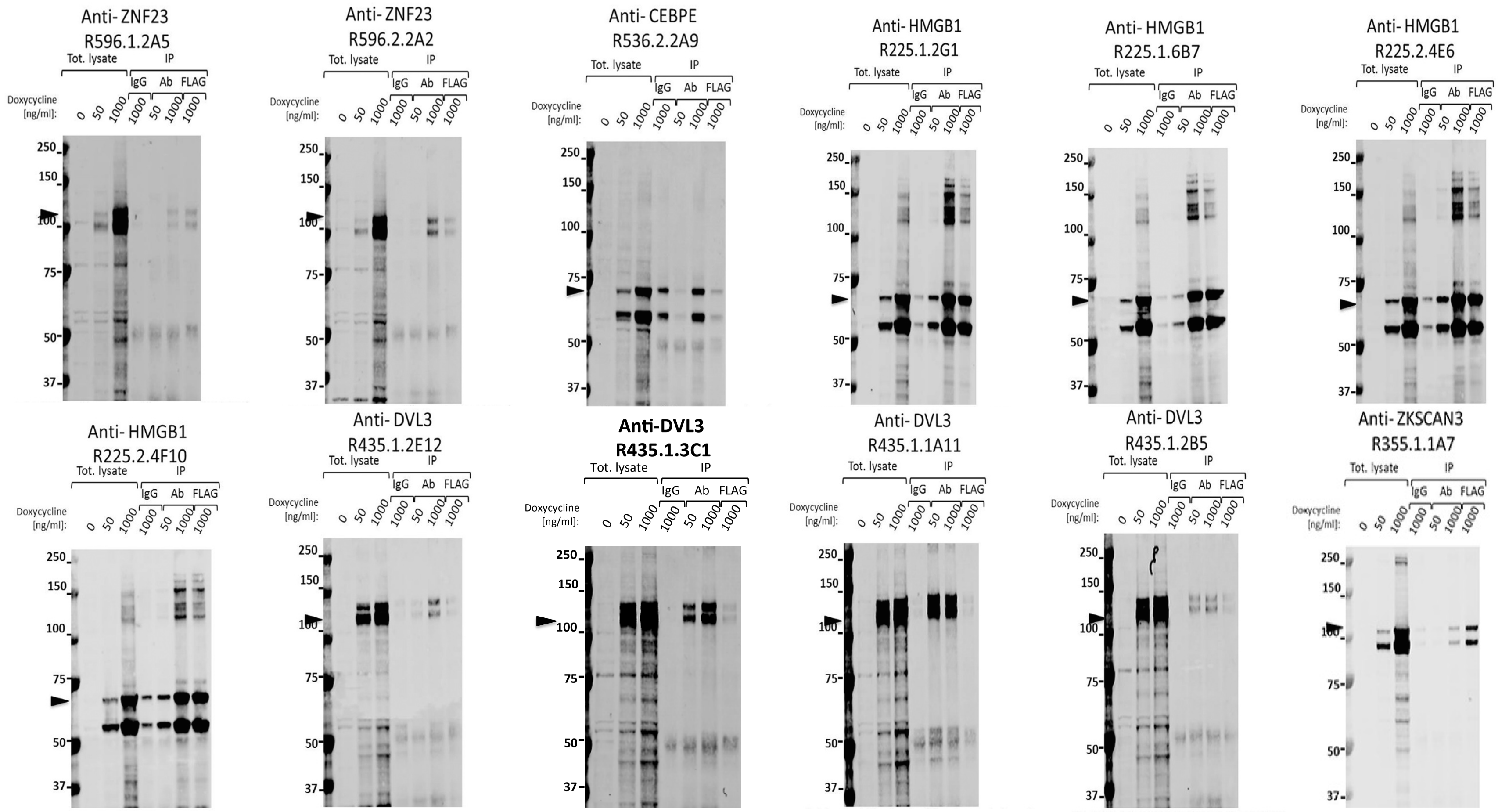
Anti- ZNF23
R596.1.2A5
Anti- ZNF23
R596.2.2A2
Anti- CEBPE
R536.2.2A9
Anti- HMGB1
R225.1.2G1
Anti- HMGB1
R225.1.6B7
Anti- HMGB1
R225.2.4E6
Anti- HMGB1
R225.2.4F10
Anti- DVL3
R435.1.2E12
Anti-DVL3
R435.1.3C1
Anti- DVL3
R435.1.1A11
Anti- DVL3
R435.1.2B5
Anti- ZKSCAN3
R355.1.1A7
Tot. lysate
IP
IgG
Ab
FLAG
Doxycycline [ng/ml]:
0
50
1000
250
150
100
75
50
37
